# Outcome analysis of holmium laser and pneumatic lithotripsy in the endoscopic management of lower ureteric calculus in pediatric patients: a prospective study

**DOI:** 10.1590/S1677-5538.IBJU.2016.0211

**Published:** 2016

**Authors:** Ankur Jhanwar, Ankur Bansal, Satyanarayan Sankhwar, Manoj Kumar, Gautam Kanodia, Gaurav Prakash

**Affiliations:** 1 King George Medical University, Lucknow, Uttar Pradesh, India

**Keywords:** Lithotripsy, Laser, Ureteroscopy, Pediatrics

## Abstract

**Objective:**

To analyse outcomes of holmium laser and pneumatic lithotripsy in treatment of lower ureteric calculus in pediatric patients.

**Materials and methods:**

Prospective study conducted between August 2013 and July 2015. Inclusion criteria were lower ureteric calculus with stone size ≤1.5cms. Exclusion criteria were other than lower ureteric calculus, stone size ≥1.5cms, congenital renal anomalies, previous ureteral stone surgery. Patients were divided into two groups. Group A underwent pneumatic and group B underwent laser lithotripsy procedure. Patient's baseline demographic and peri-operative data were recorded and analysed. Post operatively X-ray/ultrasound KUB (Kidney, ureter and bladder) was performed to assess stone free status.

**Results:**

A total of 76 patients who met the inclusion criteria to ureteroscopic intracorporeal lithotripsy were included. Group A and B included 38 patients in each. Mean age was 12.5±2.49 in Group A and 11.97±2.74 years in Group B respectively (p=0.38). Overall success rate was 94.73% in Group A and 100% in Group B, respectively (p=0.87).

**Conclusion:**

Holmium Laser lithotripsy is as efficacious as pneumatic lithotripsy and can be used safely for the endoscopic management of lower ureteric calculus in pediatric patients. However, holmium laser requires more expertise and it is a costly alternative.

## INTRODUCTION

The incidence of stone disease in the pediatric age group is on the rise particularly in developing nations like India. Management of ureteral stone in pediatric age group is often challenging. Open ureterolithotomy was the preferred treatment for ureteral stone before 1980's (1). With the improvement in surgical skills and technological advancement of the endoscopic instruments, the management of ureteral stones in children is becoming more similar to that in adults, and it has changed from more invasive open surgeries to less or minimal invasive endoscopic lithotripsy. Pneumatic and laser lithotriptors are most preferred and frequently used in intracorporeal lithotripsy during endoscopic management of ureteral stone (2). Holmium Laser lithotripsy now gained popularity and is established as standard modality (3). With the introduction of holmium YAG laser in the urological armamentarium indications for ureteroscopic stone managements have extended and now it is possible for the urologist to also manage larger stone sizes (4).

## MATERIAL AND METHODS

This prospective study was conducted after obtaining ethical review board committee approval and an informed written consent was signed from all the included patients/parents/guardians in the department of Urology of a tertiary care teaching institute situated in north India from Aug 2013 to July 2015. A total of 76 patients of 6–17 years of age met the inclusion criteria of having lower ureteric calculus (≤1.5cms) as evident by symptoms and radiological investigation (X-ray KUB/Ultrasound KUB/ Computed tomography of KUB (Kidney, ureter, and bladder region). Exclusion criteria were other than lower ureteric calculus, stone size ≥1.5cms, congenital renal anomalies, history of ureteral stone operation, active urinary tract infection, spinal deformities, bleeding diathesis.

Prior to intervention patients underwent complete physical examination, urine routine with culture and sensitivity, and blood investigation. The site and size of ureteral calculus was noted. Appropriate antibiotic was administered pre and post intervention. Patients were allocated into either group A or group B in 1:1 ratio. Group A patients underwent pneumatic lithotripsy and group B underwent holmium laser lithotripsy. Patients were followed with X-ray KUB/USG KUB region and note of complaint if any. Operating time was calculated from insertion of pediatric cystoscope into meatus to the removal of ureteroscope out of meatus.

### Technique

Both the ureteroscopic procedures were done under general anaesthesia in lithotomy position. A rigid cystoscopy was performed to locate ureteric orifice and advancement of hydrophilic guidewire under fluoroscopic guidance into the renal pelvis or beyond the level of calculus. Ureteral orifice was dilated with a balloon catheter (whenever indicated). A 6 to 7.5F semirigid ureteroscope (Karl Storz) was used for ureteroscopic lithotripsy. Post operatively fluoroscopy was performed for reassessing any residual and position of double J stents. Ureteroscopy performed, ureteroscope placed distal to stone, the holmium laser fibre (365µm) pulse frequency: 8–10Hz, and power supply: 9.6–16W was used in group B and Swiss lithoclast 2 device (Wolf) with 3F pneumatic probe was used in group A for lithotripsy. Stone was fragmented and retrieved and very tiny fragmentes were left for spontaneous passage. Double J stent was inserted in all patients. Foley catheter was placed post operatively. On postoperative day 2, stone-free state was checked with KUB films. Impacted calculus was defined as stone which did not change its position for at least 3 months.

### Statistical analysis

The results are presented in mean±standard deviation (SD) and percentages. The unpaired t-test was used to compare two independent means. The p-value <0.05 was considered statistically significant. All the analysis was carried out using SPSS 16.0 versions (Chicago, Inc., USA).

## RESULTS

Baseline demographics were comparable in both the groups (Table-1). A total of 76 (male: 66, female: 10) patients who met the inclusion criteria for endoscopic intracorporeal lithotripsy were included in the study. Group A and B both consisted of 38 patients each. Mean age was 12.5±2.49 years in Group A and 11.97±2.74 years in Group B respectively (p=0.38). In laser lithotripsy group 28 patients had right and 10 had left ueretric calculus while in pneumatic lithotripsy group 26 patients had right and 12 had left ureteric calculus. Stone surface areas were 8±3.09mm^2^ in group A and 8.2±3mm^2^ in the in group B respectively (p=0.77). Total operative times in group A was 37.13±5.94 min and in group B was 40.15±5.5 min respectively (p=0.023). Mean per urethral catheter duration in both groups was 12 hrs following endoscopic lithotripsies. Lengths of the hospital stay in group A was 2.45±0.49 and 2.27±0.43 days in group B (p=0.09). Two patients (5.62%) in group A and 3 patients (7.89%) in group B had fever (≥38.5ºC) which was managed conservatively. None of our patients experienced any major complication related to the procedure. Stone migration was observed in 2 (5.62%) patients in group A (Table-2). Antibiotic was given according to urine culture sensitivity for one day before and after procedure.

**Table 1:** Baseline characteristics of patients in both groups.

Parameters	Group A (Pneumatic)	Group B ( Laser)	p value
No. of patients	38	38	1
Mean Age±SD	12.5±2.49	11.97±2.74	0.38
Male/Female ratio	34/4	32/6	
Laterality (R/L)	26/12	28/10	
Stone burden (in mm^2^)	8±3.09	8.2±3	0.77
Prior intervention	Nil	Nil	NA
Impacted Calculus	2(5.2%)	3(7.89%)	1

**Table 2:** Perioperative clinical data of patients in both groups.

Perioperative variables		Group A (Pneumatic)	Group B (Laser)	p value
Total operative time in (mins)		37.13±5.9	40.15±5.5	0.023
Foley catheter indwelling time in hours		12	12	1
Length of hospital stay(in days)		2.45±0.49	2.27±0.43	0.09
Double J stent		38	38	1
**Complication**				
	Stone migration	2(5.2%)	0	0.49
	Fever(≥38.5°C)	2(5.2%)	3(7.89%)	1
	Ureteric injury	0	0	
ESWL (auxillary Procedure)		2(5.2%)	0	0.49
Overall success rate (n%)		94.73%	100%	0.87

## DISCUSSION

Lower ureteric calculus is not uncommon in pediatric age group. Different treatment modalities are available for the management of lower ureteric calculus for pediatric patients including ESWL (extracorporeal shock wave lithotripsy), ureteroscopy, percutaneous antegrade ureteroscopy, laparoscopic and open surgery (5). With the improvement in surgical skills and technical advancement in the working instruments (smaller calibre semi-rigid and flexible ureteroscopes), the management of lower ureteric calculus has changed from open surgery to minimal invasive endoscopic lithotripsies (6). In the current era, ureteroscopy has become the preferred modality of managing lower ureteric calculus with success rate approaching to 100% in both adults as well as in pediatric patients.

Young in 1912 was the first to perform ureteroscopy, inserted a cystoscope in a child with posterior urethral valve (7). Goodman in 1977 was the first to performed rigid ureteroscopy (8).

Different lithotriptors can be used for intracorporeal lithotripsy including electrohydraulic (EHL), ballistic (pneumatic), ultrasonic (US), laser (Ho: YAG). In the last few years lasers have been increasingly replacing others for intracorporeal lithotripsy (9, 10).

European Association of Urology (EAU) recommends Holmium YAG laser as gold standard procedure for intracorporeal lithotripsy (5). The reason behind is, its advantageous property of breaking all type of stone irrespective of their composition as compared to other lithotriptors and because of weaker shock waves there is lower risk of stone migration (11).

Pneumatic lithotripsy was first introduced into practice in 1992 in Switzerland (12). Advantage of pneumatic lithotripter when compared to other lithotriptors is its lower risk of perforating ureter and no thermal damage (13). Only concern with pneumatic lithotripter is stone migration, that ranges between 1.6% and 17.3% particularly with upper ureteral calculus (14, 15).

In the present study stone-free rate for lower ureteric calculus with holmium laser was 100% and 94.73% with pneumatic lithotripsy respectively (p=0.87). This study confirms previous reports in literature about efficacy of ureteroscopic Ho: YAG laser lithotripsy in treating distal ureteric stones (16, 17). Total operative time in this study was 40.15±5.55 min in laser lithotripsy while 37.13±5.94 min in pneumatic lithotripsy (p=0.023). In this study we observe 100% success rate with laser lithotripter and 94.73% with pneumatic lithotripsy. Similarly, Salvado et al. (18) also reported 96% success rate of laser lithotripsy in the management of distal ureteral stone. Manohar et al. (19) reported 84% success rate with laser lithotripsy. Our overall success rate for lower ureteric calculus in pediatric age group approached 97% with the ureteroscopic procedure which was in accordance with the literature.

In our study we observed stone migration into the collecting system of two patients (5.2%) in group A patients (impacted calculus), which later was managed with ESWL (after 3 days), while none of our patients in group B experienced similar complication. Razzaghi et al. (2) reported higher incidence of stone migration into renal collecting system with pneumatic lithotripter (17.9%) particularly with upper ureteric calculus and no such complication in the laser group. Salvado et al. (18) reported statistically insignificant difference of stone migration between the two modalities of lithotripsy. Similarly, Manohar et al. (19) did not observed any statistically significant difference of stone migration rates between pneumatic and laser lithotripsy groups. This is because of the improvement in surgical skills and technological advancement.

In this study, we retrogradely inserted Double J stent in all patients. In our belief ureteral stenting with double J stent prevents postoperative sepsis, and ureteral mucosal edema, although there was no clear precise indication for ureteral stenting such as ureteral injury or perforation. However, there are several prospective randomized controlled trails comparing stented versus non stented ureteroscopic lithotripsy and they reported similar outcomes (20). In the present study, length of hospital stay was comparable and we did not found any statistically significant difference between the two groups (p=0.09). This might be because none of our patients in the study had suffered any major complication related to procedure. Some studies in the literature reported complication rate of ureteroscopy between 9–25%. However, incidence of major complication is less than 0.1% n (5). Stone analysis in our study showed ammonium acid urate and uric acid stones the predominant variety. The major advantage with the holmium laser lithotripsy when compared with pneumatic lithotripter is that laser lithotripter will fragment any stone irrespective of composition.

## CONCLUSIONS

Both holmium laser and pneumatic lithotripsy are equally efficacious and can be used safely for the endoscopic management of lower ureteric calculus in pediatric patients. However, holmium laser requires more expertise and it is a costly alternative and comparatively more advantageous in impacted calculus.
